# Gecko-Inspired Controllable Adhesive: Structure, Fabrication, and Application

**DOI:** 10.3390/biomimetics9030149

**Published:** 2024-03-01

**Authors:** Yanwei Liu, Hao Wang, Jiangchao Li, Pengyang Li, Shujuan Li

**Affiliations:** 1 Key Laboratory of NC Machine Tools and Integrated Manufacturing Equipment of the Ministry of Education, Xi’an University of Technology, Xi’an 710048, China; 2 School of Mechanical and Precision Instrument Engineering, Xi’an University of Technology, Xi’an 710048, China

**Keywords:** gecko adhesion, bio-inspired controllable adhesive, biomimetic, climbing robot

## Abstract

The gecko can achieve flexible climbing on various vertical walls and even ceilings, which is closely related to its unique foot adhesion system. In the past two decades, the mechanism of the gecko adhesion system has been studied in-depth, and a verity of gecko-inspired adhesives have been proposed. In addition to its strong adhesion, its easy detachment is also the key to achieving efficient climbing locomotion for geckos. A similar controllable adhesion characteristic is also key to the research into artificial gecko-inspired adhesives. In this paper, the structures, fabrication methods, and applications of gecko-inspired controllable adhesives are summarized for future reference in adhesive development. Firstly, the controllable adhesion mechanism of geckos is introduced. Then, the control mechanism, adhesion performance, and preparation methods of gecko-inspired controllable adhesives are described. Subsequently, various successful applications of gecko-inspired controllable adhesives are presented. Finally, future challenges and opportunities to develop gecko-inspired controllable adhesive are presented.

## 1. Introduction

Since ancient times, the natural world has been the source of various technological ideas, engineering principles, and major inventions for human beings. For example, frogs have highly regular toe pad microstructures that allow them to climb on slippery surfaces like moist leaves or tree trunks [1,2,3], octopuses can capture prey of various sizes stably underwater [4,5,6], and mussels can adhere to rocks [7,8,9]. By gaining an in-depth understanding of the physiological characteristics of these organisms, researchers have developed synthetic adhesives with unique properties, which enable them to replicate the surface characteristics of living organisms [10].

In the animal kingdom, geckos are renowned for their excellent climbing ability. They can crawl or run effortlessly on the ground, walls, and ceilings. However, it was only a decade ago that the extraordinary climbing ability of geckos was revealed. Geckos can achieve adhesion and detachment of their feet to the surface in a matter of milliseconds, a capability known as controllable adhesion. Since the remarkable controllable adhesion ability of gecko feet was discovered, scientists have extensively studied their microstructure and controllable adhesion mechanism. Drawing inspiration from the structure and controllable adhesion mechanism of gecko feet, researchers have developed imitation-gecko controllable adhesives and conducted extensive research on the key factors affecting their controllable adhesion performance. Despite the significant research achievements made by scientists in this highly innovative field, bio-inspired controllable adhesives still face many challenges and unresolved issues.

In order to further promote the research of gecko-inspired controllable adhesives and improve their practical applicability, the latest progress in gecko-inspired controllable adhesives, along with their application, is reviewed. First, the adhesion mechanism of the gecko’s feet is introduced from three aspects: the structure of the gecko’s feet, the source of the adhesion force, and the behavior of the gecko’s adhesion system in the process of attachment and detachment. Then, design methods of gecko-inspired controllable adhesives based on shear adhesion are summarized from the perspective of structure, and their adhesion performances are summarized. Later, active controllable adhesion strategies based on SMP (shape memory polymers) microstructures, magnetic microstructures, and controllable back layers are introduced, and the representative fabrication methods of the gecko-inspired controllable adhesive including photolithography, ultraprecision machining, and 3D (three dimensional) printing are presented. After that, the applications of gecko-inspired controllable adhesives in climbing robots and gecko grippers are demonstrated. Finally, the future development direction of gecko-inspired controllable adhesives is predicted.

## 2. Adhesion Mechanism of Gecko’s Feet

### 2.1. The Structure of Gecko’s Feet and the Sources of Adhesion

**Figure 1:**
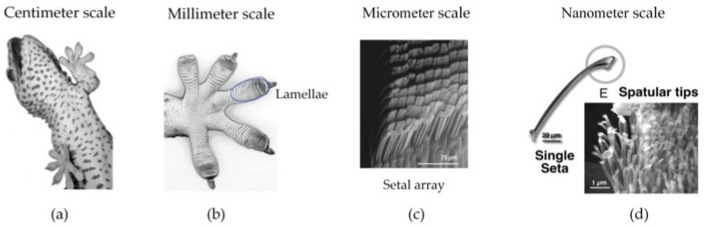
Gecko adhesive structures (reprinted with permission from Ref. [14]. Copyright 2006, Springer-Verlag). (**a**) The body of the gecko is usually in the centimeter range. (**b**) Lamellae structures on gecko paws, usually in the millimeter range. (**c**) Lamellae structures with micrometer-scale arrays of seta. (**d**) The ends of the seta have nanometer-scale spatulas.

Understanding the source of gecko foot adhesion is the key to developing a gecko-like adhesive material. With the invention of the scanning electron microscope (SEM), researchers have observed dozens of rows of lamellar structures on the toes of geckos, each consisting of thousands of setae [11]. Each seta is approximately 30–130 um in length [12] and 5 um in diameter [11]. At the end of the setae, there are about 100–1000 spatulas [12]. The length of a single spatula is about 300 nm, and the spatula ends with a flat tip that is about 280 nm at its widest point [11,13]. This multi-scale adhesive structure of the gecko from centimeters to nanometers allows the gecko paw to be in close contact with the wall, which gives the gecko an excellent climbing ability. The multi-scale composite adhesive structure of the gecko is shown in Figure 1.

Adhesion is the force of attraction created by the close contact of two different materials. There are various theories to explain adhesion such as electrostatic attraction, microinterlocking, adhesion secretion, suction, etc. As a result, the source of adhesion in geckos has been the subject of much controversy Attempts aimed at determining the source of adhesion in geckos date back to the 19th century. Initially, a number of hypotheses were proposed to explain the source of gecko adhesion such as suction, friction, and micro-interlocking [15,16,17], but none of them were supported by experimental observations.

With the development of science and technology and many experiments by researchers, it has been found that the adhesion of geckos may be an intermolecular interaction [18]. Autumn et al. used the method of micro-mechanical biaxial resistive cantilever beams to measure the force behavior of a single gecko hair in the adhesion process for the first time. The measurement results showed that the adhesion force when gecko hairs slide parallel to the wall and peel off vertically from the wall was within the range predicted by the van der Waals force model, supporting the hypothesis that the adhesion force of geckos originates from van der Waals forces [19]. Subsequently, Autumn also tested the adhesion force of individual gecko toes and single gecko hairs on hydrophobic and hydrophilic semiconductors, confirming that the adhesion force of geckos is determined by van der Waals forces, and not capillary force [20].

Although the experimental results of the gecko’s adhesion to the surface by Autumn verified that the main adhesion force between the gecko and the surface came from the van der Waals forces between molecules, they did not indicate whether humidity affected the adhesion force. Huber et al. tested the adhesion force of a single gecko seta on substrates with different hydrophobicities and at different air humidities. The results indicate that lower humidity and hydrophobicity can enhance the adhesion force of gecko setae, and when the humidity is high, the adhesion force significantly decreases [21]. However, Sun et al. found that when the relative humidity (RH) was greater than 16%, capillary force became the primary source of adhesion force. As the relative humidity increased, the adhesion force also increased [22]. Kim et al. further found through experiments that when the contact angle between the setae and the surface was 10°, the adhesion force increased with increasing relative humidity, and when the contact angle increased, the adhesion force decreased with increasing relative humidity [23]. Puthoff et al. found that with increasing humidity, the increase in adhesion force on hydrophobic and hydrophilic surfaces was similar, and even at high shear rates, the adhesion force increased with increasing relative humidity. This is because the increase in relative humidity softens the gecko setae, increasing the viscoelastic damping of the setae, thereby increasing the adhesion force, which is inconsistent with the assumption that capillary force is the main source of the gecko adhesion force [24]. Pesika et al. believe that when the setae are exposed to water, changes in the surface hydrophilic equilibrium of the setae will result in a change in the conformation of surface proteins, thereby increasing the surface energy and thus increasing the adhesion force [25].

In addition to this, some researchers have suggested that electrostatic force is the main source of gecko adhesion [26]. Lzadi et al. [27] showed that contact electrification contributed to the adhesion of geckos by measuring the magnitude of the charge and the adhesion force generated by the gecko paws when they came in contact with different materials. The measurements showed that the strength of electrostatic adhesion generated by gecko paws on Teflon AF was higher than that on the PDMS surface, so they concluded that it was CE-driven electrostatic interactions that dictated the strength of gecko adhesion. Later, Song et al. [28] proposed that the electrostatic force only accounted for 3% of the gecko adhesion force through experimental and modeling analysis. Thus, the low contribution of electrostatic force explains the movement of geckos on Teflon through the fact that although they can easily generate frictional charges, it is difficult to generate van der Waals forces. Singla et al. used interface-sensitive spectroscopy to study the contact interfaces between sapphire and bristles with and without unbound lipids. By observing the distance moved by the OH peak of sapphire, they concluded that the acid–base interaction between the gecko setae and the hydroxylated sapphire surface was the main source of gecko adhesion [29]. In addition, they mentioned a strong correlation between the acid–base properties of the material and its ability to generate charge, which supports the hypothesis that electrostatic forces enhance adhesion [29].

**Figure 2:**
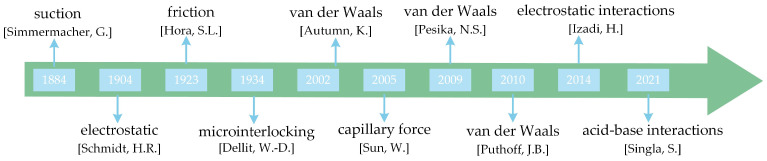
The process of exploring the sources of gecko adhesion.: suction [15]; electrostatic [26]; friction [16]; microinterlocking [17]; van der Waals (Autumn, K.) [19]; capillary force [22]; van der Waals (Pesika, N.S.) [25]; van der Waals (Puthoff, J.B.) [24]; electrostatic interactions [27]; acid-base interactions [29].

Figure 2 illustrates the process of exploring the source of gecko adhesion forces. Although there is much controversy regarding the primary source of gecko adhesion, this paper argues that the prevalent van der Waals forces are the primary source of gecko adhesion, whereas non-van der Waals forces depend primarily on the nature of the substrate, which can have an effect on adhesion. For example, capillary forces have an effect on adhesion on wet surfaces. Therefore, van der Waals forces, as the main source of gecko adhesion, is more in line with the biological properties of geckos.

### 2.2. Gecko Controllable Adhesion Mechanism

#### 2.2.1. High Adhesion Strength

Artificial adhesives inspired by geckos have essential applications in wall-climbing robots, grippers, and adhesive devices for space debris [30,31,32]. Therefore, an in-depth understanding of the mechanism of gecko-inspired controllable adhesives can help people better research the design, preparation, and performance of gecko-mimicking controllable adhesives.

The gecko can easily hang upside down on the wall using just one toe [33]. This strong adhesion ability is closely related to the hierarchical adhesion system on its foot. The main component of gecko setae is similar to bird feathers and human nails, both made of β-keratin with a Young’s modulus of 2 Gpa [34,35,36,37]. According to the Dahlquist criterion, β-keratin cannot achieve adhesion [38,39]. Therefore, human nails also cannot adhere to walls. However, geckos continuously divide their toes into thin sheets, stiff hairs, and a scalpel-like layered fiber structure, reducing the equivalent elastic modulus of their adhesion system to below 100 kPa, increasing the contact area with rough surfaces and achieving adhesion [34].

**Figure 3:**
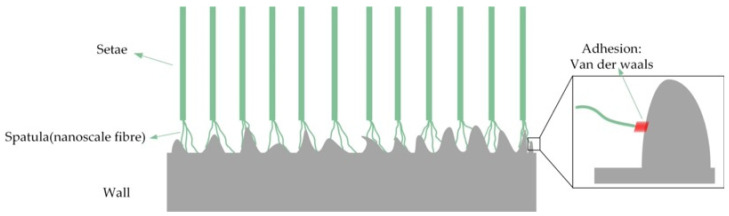
Nanoscale spatula contacting the wall.

As shown in Figure 3, when in contact with a surface, the gecko’s toes utilize a hierarchical structure with tree-like setae and spatula structures to achieve contact splitting. Subsequently, millions of nanoscale fibers undergo “molecular contact” with the contact surface, and each contact surface generates van der Waals forces. As a universally present intermolecular force, molecular polarity and distance primarily determine the van der Waals force. Although the van der Waals forces generated between each spatula and the contact surface are very weak, geckos have a large number of nanofibers, and the combined force they produce can effectively support a gecko’s agile movement on a surface. Experiments have demonstrated that a gecko’s front two feet can produce approximately 20 N of frictional force, while each seta can produce 6.2 N of shear force, with the maximum shear force reaching 194 N [40], and geckos can produce an adhesion strength of 100 Kpa [13,34].

#### 2.2.2. Controllable Adhesion and Easy Detachment

The strong adhesive force ensures the gecko’s stable attachment to the contact surface. The gecko is able to climb flexibly on the wall, switching between stable adhesion and easy detachment. Researchers refer to this characteristic as the gecko’s controllable adhesion feature.

**Figure 4:**
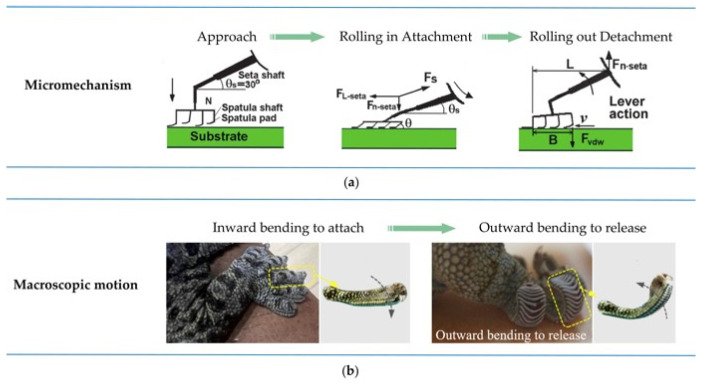
Gecko controllable adhesion mechanism. (**a**) Microscopic adhesion mechanism [44]. (**b**) Macro motion of the foot [45]. (Refs. [44,45] were adapted through open access permission).

The experimental findings revealed that when the contact angle between the gecko setae and the wall is 30°, no adhesive force is generated between the setae and the wall [41,42,43]. Tian et al. [44] established a motion model of a single seta during adhesion and detachment (as shown in Figure 4a) and analyzed the generation of gecko adhesion and detachment forces in detail. In the natural state, the spatula pad is at a 30° angle to the seta shaft and nearly perpendicular to the surface of the spatula shaft. Therefore, a weak adhesion force is generated when the spatula is almost parallelly attached to the wall. When the gecko needs to stick to the wall, its toes bend inward and shear (as shown in Figure 4b). This results in a decrease in the pull angle *θ_s_* between the setal shaft and the wall as well as the pull angle *θ* between the spatula shaft and the wall, thereby generating adhesion force. The smaller *θ_s_* and *θ* are, the stronger the adhesion force produced. During the gecko’s detachment process, the gecko’s toes bend outward, causing the setae to peel off from the wall, and intense shearing and squeezing occur between the setae and the wall, increasing the effective elastic modulus of the gecko adhesion system to above 100 Kpa, making it easy to separate from the wall. Therefore, the gecko controls the hierarchical adhesion system through the movement of its feet, and the hierarchical adhesion system closely contacts and separates from the wall under macroscopic control, thereby achieving controllable adhesion and detachment.

## 3. Gecko-Inspired Controllable Adhesive

In the early 21st century, researchers began to explore the microstructure design of gecko-inspired adhesives [46]. Van der Waals forces are the primary source of gecko adhesion and are highly sensitive to the distance between the adhesive and the wall, typically at the sub-nanometer scale [13]. Therefore, researchers have focused on the microstructure design of high aspect ratio structures [47,48], nanoscale fibers [49,50,51], and the geometric shapes of fiber ends [52,53,54,55] to enhance the adhesion of gecko-inspired adhesives.

Although the above gecko-inspired adhesive can generate a strong adhesion force, its adhesion force is not anisotropic, meaning that it cannot realize controllable adhesion. Distinguished from traditional adhesives, the controllability of adhesion force and friction force is the key feature of gecko-inspired controllable adhesive surface design, which is also the key to its application in gecko robotic adhesive feet and bionic grippers. Moreover, designing anisotropic microstructures (tilted fibers, asymmetric fibers) and using functionalized materials (shape memory polymers, adding magnetizable particles in polymers) have become the two main methods in which to make gecko-inspired controllable adhesives smarter. Therefore, this section will review the design of gecko-inspired controllable adhesives and summarize their preparation methods from these two aspects.

### 3.1. Gecko-Inspired Controllable Adhesive Based on Asymmetric Structures

Setal arrays of angled geckos are characterized by high normal compliance [56] and are vital in generating high adhesion and friction [42]. When sliding along fixed and anti-fixed directions, the array of bristles provides controlled adhesion [57]. Therefore, to achieve controlled adhesion and separation, microstructured arrays designed to be angled (anisotropic microstructured arrays) have become the preferred method for researchers to prepare controllable adhesives.

**Figure 5:**
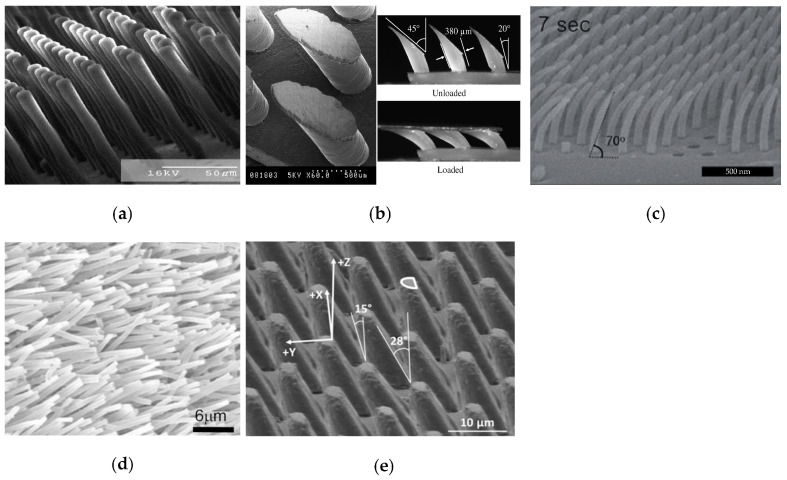
Tilted micropillar array. (**a**) Tilted micropillars without controlled adhesion properties (reprinted with permission from Ref. [58]. Copyright 2007, American Chemical Society). (**b**) Directional polymer stalks (reprinted with permission from Ref. [59]. Copyright 2012, Taylor & Francis). (**c**) Tilted high AR nanofibers (reprinted with permission from Ref. [60]. Copyright 2009, John Wiley and Sons). (**d**) Rigid polymer-polypropylene tilted microstructure arrays (reprinted with permission from Ref. [62]. Copyright 2008, American Institute of Physics). (**e**) Semi-cylindrical tilted microstructure (reprinted with permission from Ref. [63]. Copyright 2013, IOP Publishing Ltd).

In 2007, Murphy et al. first prepared tilted micropillar arrays (as shown in Figure 5a) using an angled lithography process, but they did not exhibit controlled adhesion properties [58]. Subsequently, Santos et al. [59] designed inclined micropillar structures with tilted elliptical tips, called oriented polymer stems (as shown in Figure 5b). With its tip tilted at an angle of 45° and the micropillar tilted at an angle of 20°, a 3.9 cm^2^ sample can generate an adhesion force of about 1 N when sheared along a fixed direction, and there is no adhesion force when sheared along a counter-fixed direction. Lee et al. [60] prepared tilted nanofibers with a high AR (aspect ratio) (as shown in Figure 5c) by replica imaging and post electron beam exposure, which featured the ability to time and power the electron beam exposure to control the degree of collision and bending of the nanofibers. After experimental testing, the oriented nanofibers improved the normal adhesion on soft PUA, hard PUA (polyurethane acrylate), and Teflon materials by 70%, 41%, and 129%, respectively, relative to the vertical fibers, with an anisotropy ratio of frictional adhesion of 5. The above angled microstructures were mainly prepared from soft polymers, for example, polyurethanes with a modulus of elasticity of 3 Mpa [59] and polydimethylsiloxane [61], and the microstructure arrays thus prepared are prone to collapse and self-adhesion phenomena, which affect the adhesion performance. Therefore, inclined micropillar arrays (as shown in Figure 5d) were prepared using a rigid polymer polypropylene with a modulus of elasticity of 1.5 Gpa, which has high durability and self-cleaning ability and can generate anisotropic shear forces of 4.5 N/cm^2^ and 0.1 N/cm^2^ [62]. In addition to controllable adhesion and shear, the high reusability of gecko-inspired controllable adhesives is a necessary requirement for their application in fields such as wall-climbing robots. The angled semi-cylindrical microstructure array (as shown in Figure 5e) maintains high adhesion performance after 1000 consecutive cycle tests and can generate a maximum shear adhesion force of 78 Kpa with a shear adhesion anisotropy ratio of 6.2 [63].

**Figure 6:**
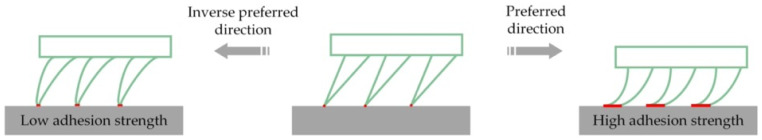
Diagram of wedge structure to realize a controllable adhesion force (vertical wedge structure as an example). The red part indicates the contact area between the adhesive and the wall.

In addition to the inclined micropillar structure, the micro-wedge structure designed by the researchers is also a typical structure to make the adhesion force anisotropic. The mechanism of the wedge structure to realize the controllable adhesion force is shown in Figure 6. When the wedge array is in initial contact with the wall, the contact area between the tip of the wedge structure and the wall is small, and the resulting adhesion force is almost negligible. When the wedge array is subjected to a shear load along the preferred direction, the wedge structure deforms and the contact area with the wall increases, resulting in an adhesion force. When sheared along the anti-preferred direction, the wedge structure is extruded against the wall and becomes more rigid, resulting in a small contact area and a weaker adhesion force.

**Figure 7:**
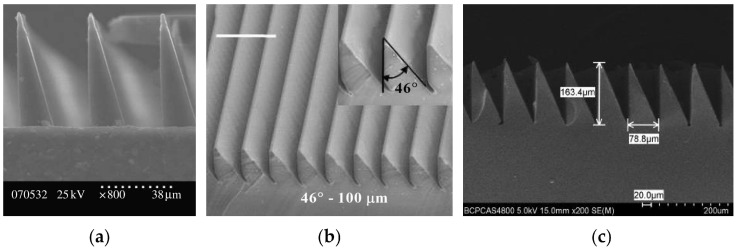
Wedge-shaped structural arrays. (**a**) Vertical wedge structures array (reprinted with permission from Ref. [64]. Copyright 2009, the Royal Society). (**b**) Array of circular wedge-shaped structures (reprinted with permission from Ref. [66]. Copyright 2017, John Wiley and Sons). (**c**) Array of wedge structures prepared on tungsten carbide molds (reprinted with permission from Ref. [68]. Copyright 2021, Tianfeng Zhou et al).

Parness et al. prepared vertical wedge microstructures (as shown in Figure 7a) using a two-step lithography process and a replica molding process with normal adhesion and shear strengths of 5.1 Kpa and 17.0 Kpa, respectively, which maintained 67% of the initial normal adhesion and 76% of the initial shear after 30,000 cycles of adhesion and de-adhesion process training [64]. To further reduce the equivalent stiffness of the microstructure array, Paul et al. used a micromachining process to fabricate a double-sided inclined wedge-shaped microstructure that could produce a normal adhesion force of 38 Kpa when a shear force of 49 Kpa was applied [65]. However, this wedge-shaped microstructure was replicated and molded based on a wax mold, which is not reusable and limits the adhesive surface’s multiple preparations. Therefore, Tao et al. [66] used an ultraprecision diamond tool to cut aluminum to form an annular metal mold (as shown in Figure 7b) that prolonged the life cycle of the mold and reduced the cost. A total of 2.84 mm^2^ of the adherent surface yielded normal adhesion and tangential shear forces of 23 mN and 125 mN in the adherence direction and 6 mN and 100 mN in the detachment direction, respectively, exhibiting prominent controlled adhesion characteristics. Capella Kerst et al. [67] fabricated copper metal molds using PDMS to replicate the wax mold cavity features and then sputtered titanium, platinum, and electroplated copper metal on a PDMS negative mold. The prepared wedge microstructures in terms of appearance and adhesion properties did not differ from those prepared from wax molds. However, the metal molds were more durable than the wax molds, did not require the molds to be sprayed with a release agent, and were easy to clean. Since aluminum is prone to thermal deformation during ultraprecision cutting, which affects the machining accuracy of the wedge structure, Zhou et al. plated Ni-P on the surface of tungsten carbide and used it as a mold to fabricate an annular wedge structure (as shown in Figure 7c) with an anisotropy ratio of each of the shear adhesion forces of 1.36 [68].

**Figure 8:**
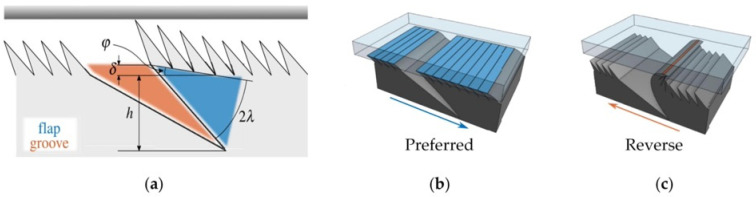
Spatially variant microstructured adhesive with one-way friction (reprinted with permission from Ref. [71]. Copyright 2019, Srinivasan A. Suresh et al). (**a**) Definition of parameters for one-way adhesive geometry. (**b**) Application of a shear force in the preferred direction results in the flap deforming to conform to the surface, yielding a large contact area (blue). (**c**) The tallest wedge at the tip of the flap prevents any other wedge from contacting the surface, reducing the contact area (orange).

The several above-mentioned controllable adhesives with wedge structures are only a collection of arrays of wedge structures with a single size feature, and some researchers have mentioned that there are also shorter bristles located closer to the thin plate and longer bristles located farther away from the thin plate in the gecko’s adhesion system [69,70]. Therefore, Suresh et al. [71] designed an array of wedge structures containing spatial variations (shown in Figure 8a), in which the height of the wedges is varied, with the tallest wedge located at the edge of the deep groove. When sheared in the preferred direction, this wedge array behaves similarly to previous homogeneous wedge features (as shown in Figure 8b). When sheared in the opposite direction, as shown in Figure 8c, the tallest wedge contacts the wall first and deforms, resulting in adhesion. Since it is higher than the rest of the wedges, it prevents the rest of the wedge structure from contacting the wall. After experimental testing, although the anisotropy ratio of the shear adhesion force could be up to 100, the value of the adhesion force was not as high as that of the adhesion surface characterized by the uniformity wedge.

**Figure 9:**
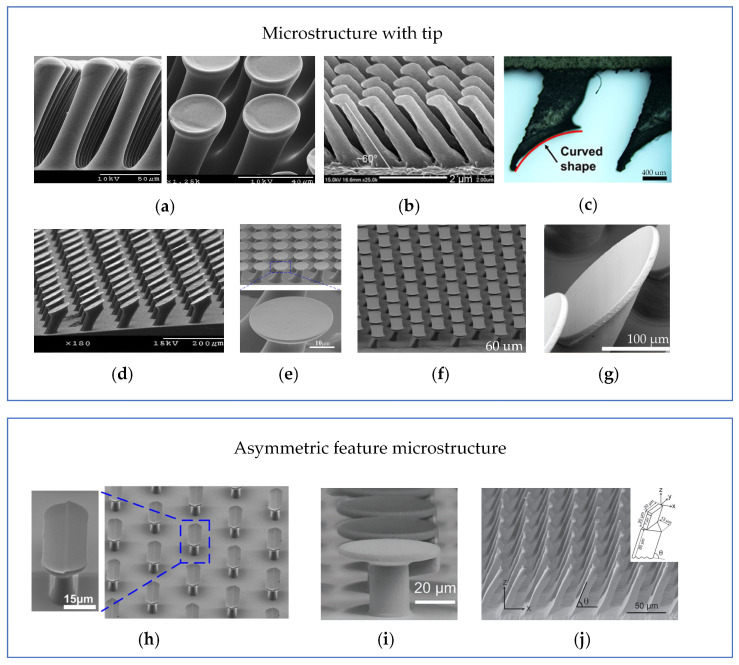
Tip-modified adhesive. (**a**) Inclined micropillar structures with spherical and spade tips (reprinted with permission from Ref. [72]. Copyright 2012, Taylor & Francis). (**b**) Nanoscale tilted microstructures with flat tips [73]. (**c**) Impregnation process modifies microstructures prepared by 3D printing (reprinted with permission from Ref. [74]. Copyright 2021, Springer Nature). (**d**) Tilted mushroom-shaped tip with modified impregnation process (reprinted with permission from Ref. [78]. Copyright 2009, John Wiley and Sons). (**e**) Tilted mushroom structure (reprinted with permission from Ref. [79]. Copyright 2014, American Chemical Society). (**f**) Rectangular cap tip structure (reprinted with permission from Ref. [80]. Copyright 2015, Yue Wang et al). (**g**) Inclined mushroom tip structure (reprinted with permission from Ref. [81]. Copyright 2020, Elsevier B.V.). (**h**) Stepped mushroom structure (reprinted with permission from Ref. [82]. Copyright 2016, American Chemical Society). (**i**) Mushroom-like structures containing TPS in the stem (reprinted with permission from Ref. [83]. Copyright 2023, Chohei Pang et al). (**j**) Inclined triangular prism with rectangular tip (reprinted with permission from Ref. [84]. Copyright 2013, John Wiley and Sons). (Ref. [73] was adapted through open access permission).

The wedge feature provides considerable controlled adhesion properties to gecko-inspired adhesives. However, it cannot generate sufficient adhesion force. It has been shown that adding tips to microstructures can enhance the adhesion force of the adherent surface. Murphy et al. [72] modified tilted polyurethane microstructures using a tip impregnation process to obtain tilted microstructures with spherical and spade tips (as shown in Figure 9a), and in comparison to the unmodified adherent surfaces, the normal adhesion force of the spherical and spade tip samples increased by a factor of 10 and 20 times, and the shear adhesion increased by 1.6 and 4.7 times, respectively. Jeong et al. [73] prepared nanoscale tilted microstructures (as shown in Figure 9b) with a tip diameter of 250 nm, a maximum shear force of 26 N/cm^2^ in the direction of adhesion, and a shear anisotropy ratio of about 11.8 using an isotropic etch and replica molding method. Then, Gwon et al. improved the adhesion performance of the adhesive surface of the inclined structure prepared by 3D printing through an impregnation process (as shown in Figure 9c). The maximum adhesion force of the adhesive surface with the added silica gel coating was increased by a factor of 4 compared to the unadded one [74]. However, the adhesion force was still minimal, only 1.53 Kpa. Experimental results have demonstrated that mushroom-shaped tips are more likely to produce higher adhesion than concave, spherical, or flat tips [75,76,77]. Therefore, Murphy modified his impregnation process, and the new impregnation process involves applying a constant load to the gecko-inspired adhesion surface during the impregnation process to prepare microstructures with an inclined mushroom-shaped tip (shown in Figure 9d), where the shear force in the adhesion direction is 5.6 times higher than that in the de-adhesion direction [78]. The impregnation process suffers from alignment and a definite lack of reflux material limitation, which leads to difficulties in accurately controlling the transverse shape and spatial consistency of the large-area tip [77]. Therefore, Wang et al. [79] prepared tilted mushroom-shaped microstructures (shown in Figure 9e) by maskless and mask exposure at the bottom and top of the photoresist, respectively, with a maximum shear adhesion of 8.4 N/cm^2^ in the preferred direction and an anisotropy ratio of 2.4. Subsequently, Wang et al. prepared tilted microstructures with a rectangular cap tip using the same preparation method (as shown in Figure 9f) that could generate a maximum shear adhesion force of 5.5 N/cm^2^ in the long-axis direction and an anisotropy ratio of 2.2 in the short-axis direction [80]. Considering that mask exposure requires a new set of mask sets for any preparation of adhesive for new microstructures, and only a small number of designs can be investigated in a short period, Busche et al. prepared micropillar arrays with tilted mushroom-shaped tips (shown in Figure 9g) using a two-photon polymerization 3D-printing process, which showed an anisotropy ratio of the normal adhesion force of 7.52 [81].

Besides designing tilted and controllable adhesive with tip features, some researchers have also designed adhesives with non-aligned features. For example, dividing the mushroom-shaped tip into upper and lower surfaces as shown in Figure 9h–j [82], adding a trapezoidal structure at the bottom of the mushroom-shaped tip [83], and fabricating tilted triangular prismatic microstructures with a rectangular tip [84] have achieved controllable adhesion strength.

Throughout the above-mentioned adhesion surfaces, the principle of realizing controlled adhesion is to change the shear direction of the adhesion surface, thus realizing a different contact area with the wall, so the basic principle is called shear adhesion. In order to more clearly show the difference in performance between the adhesive based on shear adhesion, Table 1 summarizes the fabrication methods, adhesion strength, and anisotropy coefficient.

### 3.2. Gecko-Inspired Controllable Adhesives Based on Active Modulation

Gecko-inspired controllable adhesives based on shear adhesion are designed to achieve strong adhesion and easy detachment by changing the shear loading direction of the adhesive surface. Meanwhile, researchers are also working on other techniques to change the adhesion state of the adhesive surface. For example, researchers have prepared adhesives that can actively regulate the adhesion forces from the materials used to prepare the adhesive. In this section, we will introduce gecko-inspired adhesive surfaces that can be actively regulated from two aspects, namely, shape memory polymers and composite materials as the preparation material.

#### 3.2.1. Adhesives with SMP Microstructures

Shape memory polymers, abbreviated as SMPs, can deform in response to external stimuli (electricity, heat, light) and be fixed in the deformed state when the stimuli are removed. If the external stimulus changes again in a specific way and with a specific pattern, it can be reversibly restored to the starting state. This deformation property of shape memory polymers is similar to the inward-bending adhesion and outward-bending peeling behavior of the gecko’s paws during the adhesion process. Therefore, researchers have attempted to use shape memory polymers to prepare controllable adhesives to obtain gecko-imitation controllable adhesives that can actively regulate the adhesion force.

**Figure 10:**
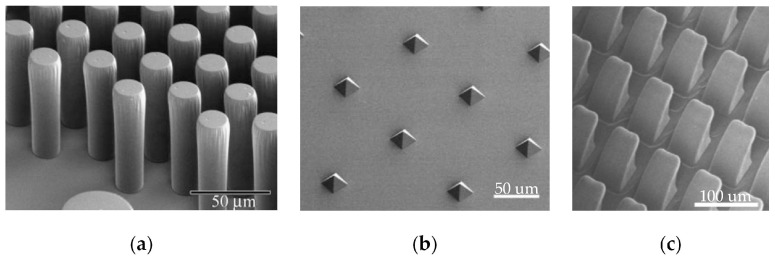
Adhesives with SMP microstructures. (**a**) Adhesive prepared by Tecoflex 72D (reprinted with permission from Ref. [85]. Copyright 2007, John Wiley and Sons). (**b**) Pyramidal microstructures (reprinted with permission from Ref. [89]. Copyright 2013, American Chemical Society). (**c**) Micro-wedge array surface of a shape memory polymer (reprinted with permission from Ref. [90]. Copyright 2016, Elsevier Ltd).

In 2007, Reddy et al. used the shape memory thermoplastic elastomer Tecoflex 72D for the first time to prepare an adhesive surface with vertical micropillar structures (as shown in Figure 10a). When the adhesive surface was heated above the transition temperature of 70 °C, the vertical micropillar structure would tilt under the pressure of the substrate surface with almost negligible adhesion. In contrast, the vertical micropillar could produce a normal adhesion strength of 29.7 Kpa. Furthermore, the authors tested the adhesion force that could be generated after the tilted micropillars were restored to vertical micropillars, which was only half that of the vertical micropillars, possibly due to the incomplete restoration of the micropillar arrays [85]. Since then, researchers have prepared controllable adhesives with vertical micropillar structures with NGDE2 [86], graphene/shape memory polymers [87], and nickel-titanium (NiTi) shape memory alloys [88], all of which exhibited actively switchable adhesion forces. Meanwhile, SMP adhesives with pyramidal microstructures have also been designed (shown in Figure 10b), which can generate a normal adhesion force of 184 N/cm^2^ on glass substrates. However, its de-adhesion force is less than 3 × 10^−3^ N/cm^2^, which exhibits a very high switchability [89]. Seo et al. combined SMPs and wedge-structured adhesives (as shown in Figure 10c) and prepared a micro-wedge array surface of a shape memory polymer that could generate a shear adhesion force in the preferred direction of 5 atm shear adhesion strength, more than three times that of the opposite direction [90].

**Figure 11:**
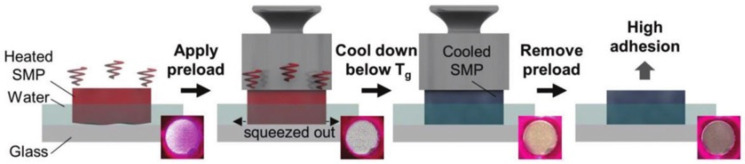
A schematic diagram of the working mechanism of an SMP underwater adhesive (reprinted with permission from Ref. [93]. Copyright 2018, John Wiley and Sons).

The adhesion force generated by the gecko-inspired controllable adhesive surface to the wall is van der Waals forces, which significantly decreases when liquid is present between them, affecting the adhesion performance of the adhesive surface and its application on some wet walls. Therefore, researchers have explored the application of shape memory polymers in preparing wet adhesives. Shao et al. made it possible for the surface to be switched between the Cassie–Baxter state and the Cassie impregnating state by depositing hydrophobic nanoparticles on adhesives prepared from shape memory polymers. In the Cassie–Baxter state, the surface had a high contact angle and low roll-off angle, exhibiting low adhesion. In the Cassie impregnating state, the surface had a high contact angle and high roll-off angle, exhibiting high adhesion. However, its adhesion decreased significantly after 25 deformation/recovery cycles due to the partial dropout of the deposited hydrophobic nanoparticles [91]. Afterward, Wang et al. combined a PU-CNF shape memory material that could be wetted with water as a substrate and an adhesion surface with a vertical microcolumn structure, which also achieved dynamic switching of adhesion to water droplets [92]. Moreover, Park et al. investigated the behavior of thermo-responsive shape memory polymers that extrude liquid when driven by pressure in the rubbery state (as shown in Figure 11). It was experimentally tested that a high strength adhesion of 17 atm could be produced, even at a slight preload of 0.25 atm when heated to 125 °C. As the preload force increased, a maximum adhesion force of 18 atm could be produced [93]. This provides guidance for improving the adhesion of gecko-inspired controllable adhesives on wet walls.

**Figure 12:**
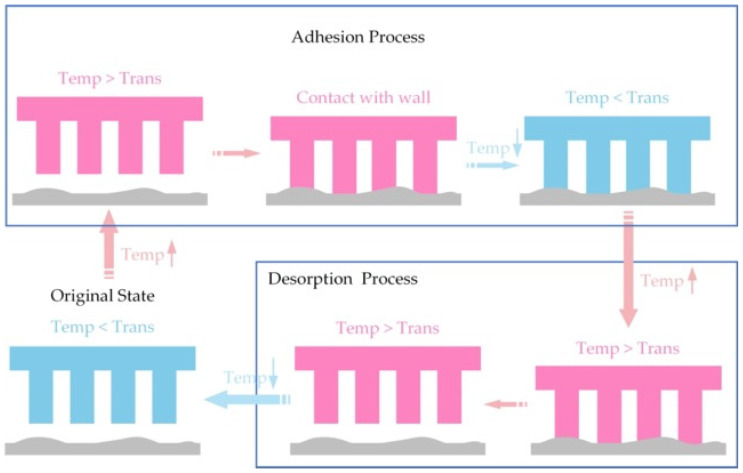
Schematic representation of the adhesion mechanism of a controllable adhesive of shape memory polymers.

The basic process described above for the shape memory polymer-based controllable adhesive surface to achieve active switching between a high adhesion state and an almost zero adhesion state can be summarized as follows. As shown in Figure 12, first, at room temperature, the adherent surface maintains its permanent shape. At the time of adhesion, the temperature of the adherent surface is raised above the transition temperature by heating. At this time, the adherent surface becomes soft and deforms under a certain pressure, creating a large contact area with the test wall. Then, the adherent surface will gradually cool down to room temperature. Its microstructure will remain temporarily fixed and have conformal contact with the wall with high adhesive force. Finally, when it is time to detach, the adherent surface is reheated above the transition temperature, and the adherent surface returns to its starting state and becomes soft, resulting in almost 0 adhesion force when detached from the wall.

#### 3.2.2. Adhesives with Magnetic Microstructures 

**Figure 13:**
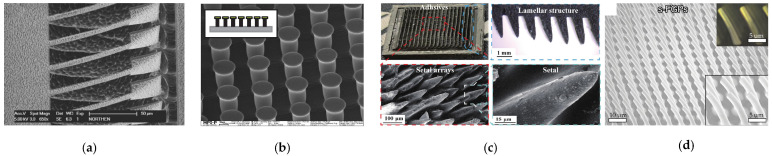
Adhesives with magnetic microstructures. (**a**) Controllable adhesive with nickel beams (reprinted with permission from Ref. [94]. Copyright 2008, John Wiley and Sons). (**b**) Magnetically actuated arrays of micropillars (reprinted with permission from Ref. [95]. Copyright 2013, John Wiley and Sons). (**c**) Adhesives composed of lamellar structures and setal arrays (reprinted with permission from Ref. [96]. Copyright 2023, Springer Nature). (**d**) Slanted functional gradient micropillars (reprinted with permission from Ref. [97]. Copyright 2018, American Chemical Society).

The deformation of shape memory polymers requires a long warming process, limiting the application of gecko-inspired controllable adhesives in fields such as wall-climbing robots. Instead, it can provide fast switching using a magnetic field. For example, Michael et al. used a combination of photoresist and nickel to prepare an adhesive surface shaped like a gecko composite structure (as shown in Figure 13a), where the composite structure with nickel cantilever beams repositioned the nickel cantilever beams under the action of a magnetic field, resulting in a lateral rotation of the end, which significantly reduced the contact area with the wall and decreased the adhesion force [94].

NdFeB particles are characterized by high saturation magnetization strength, low coercivity, and low hysteresis loss. The doping of NdFeB particles in PDMS will give the material high magnetization and easy response to changes in the external magnetic field, which is suitable for the preparation of magnetically-driven composites. Based on this principle, Dirk-Michael et al. prepared magnetic PDMS microcolumns (as shown in Figure 13b) by adding NdFeB particles with a concentration of 20% in PDMS. The adhesion force of the microarrays was 11 mN when no magnetic field was present, and it decreased to 0.7 mN in the presence of a magnetic field [95]. Shi et al. prepared a laminated wedge-shaped structured adhesive (as shown in Figure 13c) using a mixture of PDMS/NdFeB, with a switchable range of adhesion force of 40% [96].

Although the above magnetically-driven adhesive achieved rapid modulation of the adhesion force, the authors did not report on the durability of their adhesive. Wang et al. obtained novel nanocomposites by using nano-reinforcements of iron oxides modified with polyurethane acrylates, thus fabricating tilted functional-gradient pillars (s-FGPs) (as shown in Figure 13d). The shear adhesion of the s-FGPs was 9 N/cm^2^, and there was no adhesion degradation even after more than 2000 cycles of adhesion/desorption [97]. The stability of magnetorheological elastomers (MREs) can effectively mitigate the flow and deposition tendency of magnetorheological materials [98], and the controllable adhesive prepared from them showed a 2-fold increase in adhesion force. Furthermore, they could switch between strong and weak adhesion states within 1 s [99].

#### 3.2.3. Adhesives with Controllable Back Layers 

**Figure 14:**
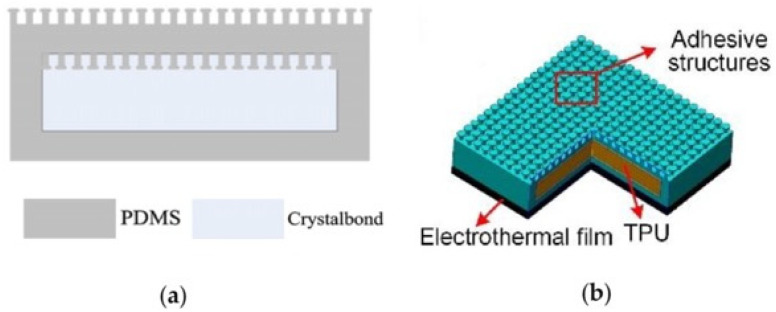
Adhesives with controllable back layers. (**a**) Adhesive with using embedded phase change material (reprinted with permission from Ref. [102]. Copyright 2010, IOP Publishing Ltd). (**b**) Hierarchal adhesive structure (reprinted with permission from Ref. [103]. Copyright 2020, American Chemical Society).

Studies have shown that adjusting the stiffness of an adhesive surface’s backing layer can affect the adhesive surface’s controllable adhesion properties. A lower stiffness of the backing layer adapts better to the wall surface and has a larger contact area at the time of contact. Moreover, the shift to higher stiffness of the backing layer can lead to a more uniform load distribution and avoid localized detachment, resulting in higher adhesion strength [100,101]. Krahn et al. embedded Crystalbond^TM^ phase change material into the backing layer of a mushroom-shaped adhesive surface (as shown in Figure 14a). The adhesion surface can thus be controlled by controlling the softness of the phase change material to control the adhesion force. During the adhesion process, the softening of the phase change material increases the contact area with the wall. Subsequent change of the phase change material from soft to hard can lead to a more uniform load distribution on individual mushroom-like fibers, thus avoiding stress concentration and improving the de-adhesion force [102]. Li et al. prepared a three-layered composite adhesion surface (as shown in Figure 14b) that used a mushroom-like adhesion layer as the top layer, a stiffness-variable layer made of thermoplastic polyurethane as the middle layer, and an electro-thermal film as the bottom layer. The electric heating film was heated by controlling its voltage. When the temperature exceeded the softening point of the thermoplastic polyurethane, the overall structural stiffness of the adhesive surface became smaller, the contact area with the non-flat wall increased, and the adhesion force could be increased by three orders of magnitude [103].

In summary, gecko-inspired controllable adhesives can be divided into two types: (1) By designing anisotropic microstructures, the adhesive will have different adhesion strengths under shear in different directions, thus realizing controllable adhesion force. (2) Controlled adhesion to the adhesive is achieved by active modulation methods such as temperature and magnetic field. 

First of all, for the first type, the advantage is that the precise control of adhesion force can be realized by designing special anisotropic microstructures, thus improving the control efficiency. However, its normal adhesion force is generally lower than normal mushroom-type microcolumns. Second, for the second type, active modulation strategies such as temperature field, magnetic field, electric field, etc. provide new ideas for switching the adhesion state of gecko-inspired adhesives and generally have very large switching ratios. However, they need to carry additional energy devices, and temperature regulation methods suffer from long regulation cycles. In addition, for adhesion surfaces with adjustable stiffness of the backing layer, the microstructure of the adhesion layer is generally characterized by mushroom-shaped microcolumns, which enhances the detachment force. Therefore, in the future, the combination of anisotropic microstructured adhesion layers with stiffness-tunable backing layers can be investigated to achieve strong adhesion and easy detachment.

## 4. Fabrication and Materials

In addition to the microstructural design of gecko-inspired controllable adhesives, the preparation method and the choice of materials are also important factors affecting the adhesion performance of gecko-inspired controllable adhesives.

### 4.1. Fabrication

Based on the microstructural design of controllable adhesives, a series of unique methods have been used to prepare these mimetic structures. Currently, the preparation methods of gecko-inspired controllable adhesives are mainly divided into two categories: the replica molding process and gas-phase growth method. The gas-phase growth method is not as widely used as the replica molding process because of the expensive experimental equipment and extremely harsh experimental conditions [104]. In addition, the surface area of the imitation gecko adhesion prepared by the gas-phase growth method is currently too small, and most are at most 1 cm^2^ [104,105,106,107,108]. Therefore, this section focuses on the research related to the replica molding process.

The replica molding process refers to the polymer material cast into the template with the micro geometric features of the gecko-inspired controllable adhesive surface, and the adhesive surface is obtained by peeling off the polymer material after curing, which is the most widely used method in the preparation of gecko-inspired controllable adhesives. Molds for casting are usually prepared using photolithography, ultraprecision machining, 3D printing, and other methods.

#### 4.1.1. Photolithography

**Figure 15:**
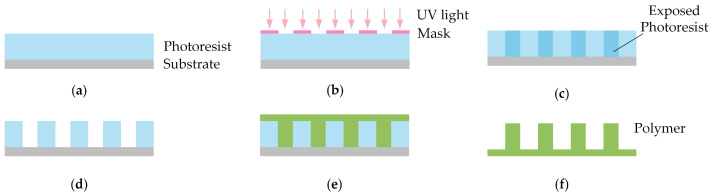
Photolithography for the preparation of gecko-inspired controllable adhesives. (**a**) The photoresist is spin-coated on the substrate. (**b**) The photoresist is exposed with a mask from the front side. (**c**) The photoresist is precisely exposed by controlling the time. (**d**) The photoresist is precisely developed by controlling the time, leaving undercut holes. (**e**) Polymer is mixed, then poured on the mold and cured. (**f**) The cured polymer is demolded, leading to the cylindrical structure.

Photolithography refers to the use of a photoresist to transfer patterns from a mask plate to a substrate (silicon wafer) under the irradiation of ultraviolet light, an electron beam, or an ion beam. Since it can precisely control the size and shape of micro/nanostructures, it is widely used to prepare gecko-inspired controllable adhesive surface molds. As shown in Figure 15, photolithography generally involves glue application, soft baking, exposure, development, and rinsing. First, photoresist in liquid form is spin-coated onto a clean substrate. Then, it is soft baked to remove solvents from the photoresist and improve the photoresist’s adhesion to the substrate. After that, selected portions of the photoresist are exposed to light sources such as UV light and electron beams. During exposure, the photoresist undergoes a series of reactions that change its physical and chemical properties. During development, the exposed or unexposed portion of the photoresist is dissolved by the developer solution so that the microstructure of the adherent surface is obtained.

**Figure 16:**
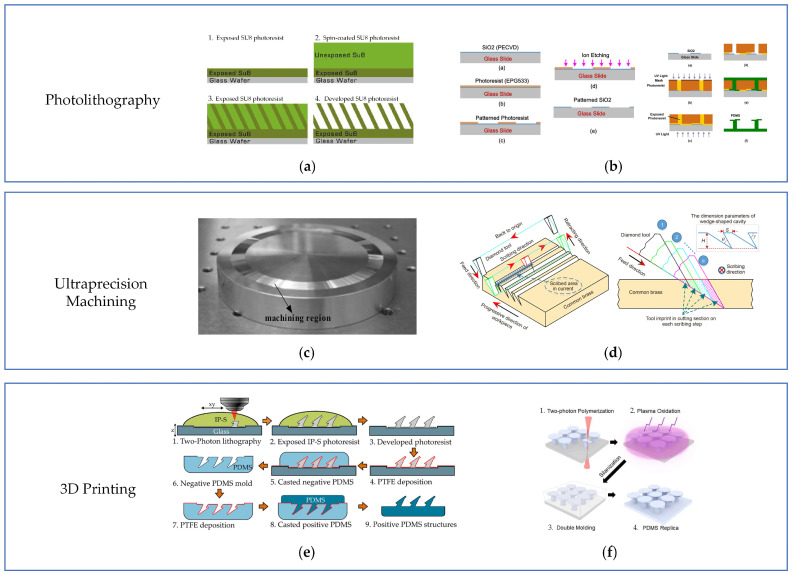
Fabrication of gecko-inspired controllable adhesives. (**a**) Fabrication process of inclined micropillar fibers (reprinted with permission from Ref. [58]. Copyright 2007, American Chemical Society). (**b**) Two-step photolithographic fabrication process for step-shaped mushroom tip adhesive (reprinted with permission from Ref. [82]. Copyright 2016, American Chemical Society). (**c**) Ring wedge metal molds made by ultraprecision diamond cutting (reprinted with permission from Ref. [66]. Copyright 2017, John Wiley and Sons). (**d**) The schematic diagram of ultraprecision multistep and layered scribing (reprinted with permission from Ref. [109]. Copyright 2021, Springer Nature) (**e**) Two-photon lithography fabricates an adhesive with tilted mushroom-like tips (reprinted with permission from Ref. [81]. Copyright 2020, Elsevier B.V.). (**f**) Two-photon lithography to fabricate mushroom-like microstructures with TPS structures (reprinted with permission from Ref. [83]. Copyright 2023, Chohei Pang et al.).

Various shapes of gecko-inspired controllable adhesives have been prepared by adjusting the exposure angle and exposure time or performing multi-step lithography. For example, Murphy et al. prepared master molds for two microcolumn structures, vertical and inclined, by spin-coating SU-8 photoresist on a glass wafer substrate and exposing it using UV light (as shown in Figure 16a). Liquid silicone rubber was then cast on the primary mold, and a flexible mold with a negative shape was obtained after curing for 24 h. Finally, polyurethane elastomers ST-1087 and ST-1060 were cast on the negative mold to obtain an adhesive surface [58]. However, this one-sided, single-tilt exposure method cannot prepare adhesives with more complex structures like mushroom shapes, so researchers developed a two-sided exposure method and two-step lithography to prepare gecko-inspired controllable adhesives with tilted mushroom-shaped structures [79], wedge-shaped structures [64], step-shaped mushroom-shaped structures [82], and semi-cylindrical microstructures [63]. For instance, Wang et al. first prepared SiO_2_ films on transparent slides using plasma-enhanced chemical vapor deposition and then fabricated EPG533 masks on SiO_2_ films using photolithography. Finally, the SiO_2_ films were structured by etching them in an ICP-CVD chamber (as shown in Figure 16b). Based on the structured SiO_2_ films, the authors prepared step-shaped mushroom-like adhesives using double-sided exposure.

Photolithography has been widely used in the preparation of bioinspired gecko-inspired controllable adhesive surface molds. However, it involves a series of complex steps and has the disadvantages of small sample size (from a few millimeters to several tens of millimeters), low yield, and high cost. Therefore, researchers have begun to explore the application of ultraprecision machining technology in preparing adhesive surface molds.

#### 4.1.2. Ultraprecision Machining Technology

In recent years, with the rapid development of ultraprecision machining technology, it has been possible to machine surfaces with an optical finish (Ra < 10 nm) and sub-micrometer geometric errors, and it has been widely used in the fields of electronics, aerospace, and medicine [110]. For example, Fresnel lens molds with a surface roughness of 7.3 have been fabricated [111], and Fresnel lens molds are available in lengths up to 2 m [66]. Therefore, ultraprecision machining technology has excellent potential for applying gecko-inspired controllable adhesives where the structural size is usually in the micro/nanoscale.

Tao et al. first prepared a circular wedge-shaped structural mold (as shown in Figure 16c) using an ultraprecision diamond-cut aluminum die. Then, they cast PDMS to obtain a gecko-inspired wedge-shaped adhesion surface [66]. Afterward, Zhou et al. used ultraprecision diamond to cut nickel-phosphorus coated tungsten carbide to fabricate a ring-shaped wedge-shaped structural mold without passing through the width, which improved the disadvantage of aluminum’s susceptibility to deformation under pressure and heat and enhanced the microstructural accuracy of the adherent surface [68]. In addition, Wang et al. proposed an ultraprecise multistep layered scribing method for machining square copper-metal molds with continuous and inclined wedge-shaped cavities. As shown in Figure 16d, multistep layered scribing utilizes a diamond tool with a V-shaped cross-section parallel to the wedge-shaped cavity to be formed. Then, the tool is made to cut the blanks along the lower surface of the wedge-shaped cavity in multiple steps, layer by layer, to form the wedge-shaped cavity, which has an average finish of Sa = (9.333 ± 0.577) nm on the lower surface of the wedge-shaped cavity [109].

#### 4.1.3. 3D Printing

3D printing is an advanced technology for rapid production by layer-by-layer prototyping with the help of computer-aided design/analysis/manufacturing. 3D printing has developed various additive manufacturing methods such as fused deposition molding (FDM), digital light processing (DLP), and two-photon polymerization (TPP) and can print metals, ceramics, polymers, and composites [112]. Since 3D printing uses a bottom-up manufacturing method and can process complex structures with high precision, it is ideally suited for the direct preparation of gecko-inspired controllable adhesives or the fabrication of molds. As shown in Figure 16e, Bushe et al. used a two-photon photolithographic to fabricate adhesive surface molds with tilted mushroom-like tip structures. To shorten the printing time, 2PP cured only the scaffold portion of the microstructure, which was developed, rinsed, and exposed for 5 min using a UV flood to maximize the stability of the structure. In order to prevent the PDMS from adhering to the mold during subsequent casting and demolding, a plasma-enhanced chemical vapor deposition method was used to deposit an octafluorocyclobutane head layer on the surface of the mold, and its anti-adhesive properties were reduced after several castings [81]. After that, Pang et al. [83] also prepared mushroom-like microstructures with a TPS structure (shown in Figure 16f) using the same method.

### 4.2. Materials

Most of the materials used to prepare gecko-inspired controllable adhesives are polymeric materials. Therefore, the physical properties of the casting material directly affect the adhesion properties of the gecko-inspired controllable adhesive. In general, the influence of several factors should be considered when selecting the casting material. Table 2 summarizes the performance parameters of common casting materials in the replica molding process.

(1) Modulus of elasticity of the material. The modulus of elasticity affects the degree of deformation of the adherent surface under the action of shear force, where the lower the modulus of elasticity, the softer the adhesive surface, as only a small preload can be generated with the wall of the contact area. However, too small a modulus of elasticity will lead to a gecko-inspired controllable adhesive of the microstructure between each other, leading to a gecko-inspired controllable adhesive surface of the self-adhesive properties of the decline, while a higher modulus of elasticity will help the self-cleaning of the adhesive surface. Therefore, materials with a suitable elastic modulus should be selected for the preparation of the adhesive surface.
(2) Material fluidity. The microstructure of the gecko-inspired controllable adhesive is usually at the micron level, so the casting material needs to have a high fluidity in order to completely fill the cavity of the mold. The index used to measure the fluidity of the material is generally the viscosity of the material; too much viscosity will lead to a slow filling process and require additional pressure to fill. At the same time, the low viscosity of most of the material does not have a strong adhesive force after curing, making it easy to separate from the mold surface.
(3) Tensile strength. The casting material needs to overcome the vacuum and the friction of the mold surface to separate from the mold after curing. Due to the micron size of the microstructure, the mold will be damaged if the casting material breaks during the demolding process and thus falls into the mold.
(4) Adhesion after curing. The adhesion of the casting material after curing and demolding also has an effect on the performance of the gecko-inspired controllable adhesive, and the adhesion strength of the material with adhesion after curing is generally higher than that of the material without adhesion.
(5) Curing type. There are two main kinds of curing type for casting materials: heating curing and room temperature curing. Heating curing requires the corresponding molds to have the nature of high temperature resistance, which affects the selection of a process for the preparation of a gecko-inspired controllable adhesion surface.

As evident from the data presented in Table 1 and Table 2, ST-1060 type polyurethane was selected as the material for creating the early-stage gecko-inspired controllable adhesive. Nonetheless, it exhibited stickiness after solidification, leading to challenges in demolding. Consequently, in recent years, Sylgard 184 and Sylgard 170, characterized by easier demolding and relatively lower modulus of elasticity and hardness, have been employed as substitutes. Sylgard 170 is prone to fracture during the demolding process because of its low tensile strength, so it is now common to use Sylgard 184, which has a higher tensile strength, as the preferred preparation material.

## 5. Applications

### 5.1. Climbing Robots

**Figure 17:**
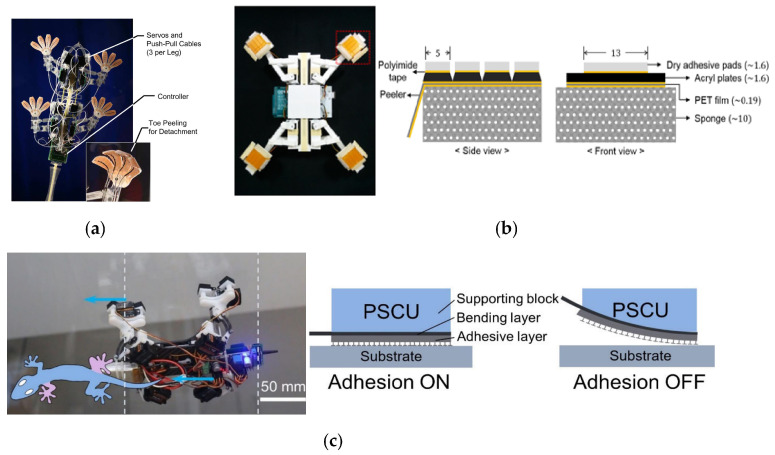
The climbing robots based on the gecko-inspired controllable adhesives. (**a**) Stickybot, a bioinspired robot capable of climbing smooth surfaces (reprinted with permission from Ref. [114]. Copyright 2008, IEEE).(**b**) Wall and ceiling climbing quadruped robot with superior water repellency (UNIclimb) (reprinted with permission from Ref. [118]. Copyright 2017, Springer Nature). (**c**) Gecko-inspired four-legged robot climbing on an inverted glass ceiling (reprinted with permission from Ref. [119]. Copyright 2021, Xiaosong LI et al.).

The ability of geckos to walk on walls has inspired researchers to study gecko adhesion mechanisms and gecko-inspired controllable adhesive materials. Attempts have been made to reproduce the same excellent wall-climbing ability as geckos in wall-climbing robots. Kim et al. fabricated Stickybot quadrupedal wall-climbing robots using previously fabricated oriented polymer shanks as the adhesion unit (as shown in Figure 17a) that are capable of climbing on a 90° of various smooth surfaces such as glass, smooth tiles, and acrylic at a speed of 4 cm/s [114]. However, its adhesive surface has no self-cleaning ability due to its large size, and it can only crawl upwards along the wall due to insufficient degrees of freedom.

Most gecko-inspired adhesives are tested in the laboratory. When applied to wall-climbing robots, there are problems with alignment and uneven loading, thus they fail to reproduce the excellent adhesion performance of the adhesive on the test bench. For example, a Stickbot robot fitted with an oriented polymer shank should be able to support a deadweight of 5 kg, but the Stickbot is only 0.3 kg [115]. Therefore, Hawkes et al. built a composite foot structure consisting of a wedge-shaped adhesive surface, a rigid tile backing, a flexible support, and tendons. The rigid tile backing helped to distribute the load uniformly over the wedge-shaped adhesion surface. The flexible support inspired by the venous blood sinus of geckos [116,117] allowed the wedge-shaped adhesion surface to be more compliant with the wall to increase the contact area and provides some preload to the rigid backing to initiate the wedge-shaped gecko-inspired adhesive surface. The tendon connected to the center of the rigid tile backing was connected to the pressurized bladder, which allowed the load to be evenly distributed across the rigid backings to maximize the adhesion performance of the entire foot, and a single adhesion surface with this composite structure could support a 0.8 kg Stickybot III and a 4 kg RiSE robot [117].

The wedge-shaped structure of the adhesive surface suffered from low normal adhesion force and insufficient design of the adhesion foot and gait, which led to the failure of the above wall-climbing robot in realizing transition climbing between the vertical wall and the ceiling. One practical solution is to use an adhesive with mushroom-like microstructures and a high adhesion strength. Ko et al. proposed a wall-climbing robot named UNIclimb that could walk on ceilings and wall surfaces with different angles. As shown in Figure 17b, its adhesion layer consists of four individually separated mushroom-like adhesives, and the separated adhesive prevents crack extension to ensure the robot’s stability for wall climbing. The robot can walk on walls at about 1.4 mm/s and can crawl on ceiling surfaces at 1 mm/s [118]. Li et al. proposed a design scheme for a soft–rigid–soft sandwich composite adhesion surface (as shown in Figure 17c), where the bending layer consisting of foam and rigid substrate will, when in a bending state, push the adhesion layer to be peeled off at a large angle, resulting in a significant reduction in the adhesion area that leads to de-adhesion, while the strong adhesion force during adhesion consists of a mushroom-like structure adhesive [119].

Existing gecko-inspired robots can realize climbing on smooth walls and ceilings, but have not yet been successfully applied in practice. The main factors that limit its practical application are as follows. First, the current gecko-inspired adhesives usually do not have a self-cleaning function, and the adaptability of the wall morphology is insufficient, thus leading to a gecko-inspired robot that can only adhere to a smooth wall surface. Second, compared with geckos, existing robots are relatively large in structural size and weight, leading to high requirements for the strength of the foot of the gecko-inspired adhesive. Finally, the gecko’s stable climbing on the wall is a result of the synergistic cooperation between the foot and the body, and the existing climbing robots have not yet considered the effect of this, resulting in their poor flexibility and climbing speed. 

### 5.2. Gecko Grippers

The end-effectors of conventional robots are mainly categorized into contact (negative pressure adsorption, magnetic adsorption, mechanical clamping) and non-contact. Negative pressure adsorption can cause damage to fragile parts, and magnetic adsorption requires specific adsorption materials. Ultrasonic technology has the advantages of high precision and low energy consumption, but it can cause the warping of precision objects. Gecko-inspired controllable adhesives with an ultra-low preload can adhere to the object’s surface, which provides a solution for robotic end-effectors to achieve the non-destructive manipulation of precision objects.

**Figure 18:**
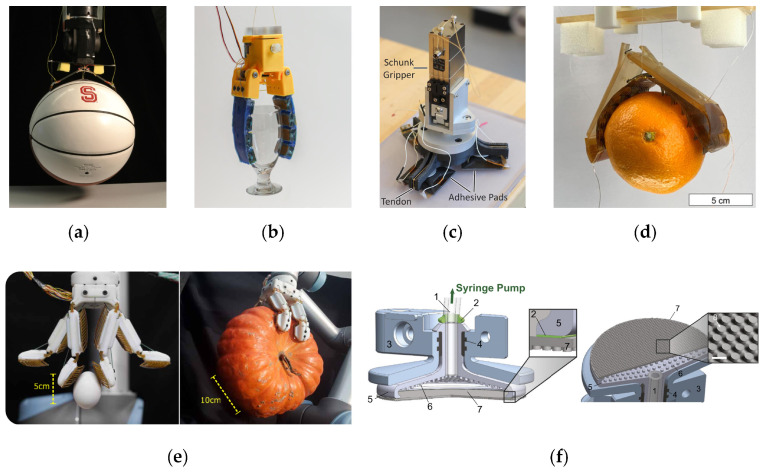
Gecko grippers. (**a**) Shear force gripper holding a regulation-size basketball (reprinted with permission from Ref. [120]. Copyright 2015, IEEE). (**b**) An electrostatic/gecko-inspired adhesives soft robotic gripper (reprinted with permission from Ref. [124]. Copyright 2020, IEEE). (**c**) Gripper with three hybrid electrostatic/gecko adhesive (reprinted with permission from Ref. [125]. Copyright 2016, IEEE). (**d**) Mechanically flexible surface structures with embedded conductive electrodes grabbing oranges (reprinted with permission from Ref. [126]. Copyright 2023, Dong Geun KIM et al). (**e**) FarmHand demonstrates its gentle, hyperextended pinch on a raw egg, a high-force power grasp on a pumpkin (reprinted with permission from Ref. [127]. Copyright 2021, Wilson Ruotolo et al). (**f**) A cross-section of 3D assembly of the system from side and from bottom of the system. 1: silicone tubing, 2: vinylsiloxane, 3: outer case, 4: rubber ring, 5: soft chamber, 6: spacer between the chamber and the FAM, 7: FAM, and 8: mushroom-shaped PDMS microfiber [128]. (Ref. [128] was adapted through open access permission).

Cutkosky et al. designed simple curved grippers containing a wedge-like gecko-inspired adhesive (as shown in Figure 18a) that conformed to the curvature of a convex object surface and used the central tendon to pull up the adhesive on both sides, thus enabling shear grasping. The authors also proposed a theoretical model that predicted a decrease in adhesion force as the radius of curvature of the object increased [120]. However, these grippers cannot apply moments, so they cannot manipulate the object at will. In many cases, the grippers need to rotate the object, and to fulfill this requirement, the authors then designed curved grippers with the ability to apply moments, where the shear force was combined with the normal pressure of the frame on the object, allowing the device to apply moments on the object [121]. Subsequently, Cutkosky et al. also designed lateral grippers that had airbags on the back of the wedge-shaped adherent surfaces, which allowed the grippers to conform to uneven surfaces, with a pair of wedge-shaped adhesives moving in opposite directions guided by linear bearings, thus lifting the object using shear force [122,123].

Gecko-inspired adhesives perform well on smooth surfaces but tend to fail on rough surfaces. Electrostatic adhesion uses a set of conductive electrodes deposited inside a dielectric. Applying a high voltage potential to the electrodes creates an electric field, which generates adhesion on both conductive and nonconductive surfaces but provides little adhesion [123]. In contrast, adhesives that combine the characteristics of electrostatic and gecko-inspired adhesives can work on smooth, slightly rough, curved, flat, conductive, and nonconductive surfaces. For example, combining a hybrid electrostatic/gecko adhesion surface with an elastic soft grip (as shown in Figure 18b) increased the gripping force on acrylic and polyimide film hemispheres with different diameters by 100% and 168%, respectively [124]. Dadkhah et al. [125] changed positive and negative electrodes, which were in the same plane, to be in different planes, which increased the adhesion’s strength by almost three times. Additionally, they designed a gripper with a three hybrid electrostatic/gecko adhesive (as shown in Figure 18c). Kim et al. designed a gripper based on shear-induced adhesion using elastomer-AgNW composite surface electrodes with different angles, shapes, and sizes as adhesion surfaces (as shown in Figure 18d). The gripper can regulate the electrostatic attraction at the contact interface by manipulating the localized electric field between the 3D flexible surface electrodes, and can grasp fruits, metals, plastics, ceramics, and other objects [126].The hybrid electrostatic/gecko adhesive utilized an electrostatic element to increase the preload of the gecko adhesive, resulting in a larger actual contact area and, thus, improved adhesion. In turn, the gecko adhesive could bring the electrostatic adhesive closer to the surface of the object due to its microstructure, resulting in deeper electric field penetration and higher electrostatic adhesion; through this positive feedback loop, the adhesion of both the single gecko adhesive and the electrostatic adhesive can be effectively improved.

In addition to these independently designed grippers based on gecko-inspired controllable adhesives, gecko-inspired controllable adhesives can also be used to enhance the gripping capabilities of existing grippers. Ruotolo et al. combined an adhesive surface with a multi-finger gripper to provide the gripper with simultaneous flexibility, stiffness, and high adhesion (as shown in Figure 18e). The backing layer of the adhesive surface is made of flexural ribs, which can undergo bending deformation in order to distribute the shear load equally and improve flexibility for the gripper [127]. The adhesion-based soft gripping system consists of an adhesion membrane and a pressure-controlled deformable gripper support (as shown in Figure 18f), which controls the adhesion strength by varying the internal pressure and the mechanics of equal load sharing at the interface. This soft-system architecture addresses the fundamental challenge of having high surface compliance while maintaining high fracture strength, thus allowing the handling of complex 3D and deformable objects [128].

As shown above, the gripping gecko gripper can grip objects with large curvature and regular shapes, but it is difficult to adapt to objects with irregular surface shapes. The soft gripper has the characteristic of adapting to complex shapes, while a composite of the gecko-inspired adhesive and soft body can improve the load capacity of the soft gripper. The combination of suction cups and adhesive surfaces can be used to grip objects with deformable surfaces.

## 6. Conclusions and Outlook

Since the discovery of the fact that the gecko’s excellent wall-climbing ability comes from the van der Waals forces generated by the microscopic setae of the paws in intimate contact with the wall surface, people have been devoted to the study of gecko-inspired dry adhesives. In this review, the microstructure of the gecko’s feet was introduced, a hierarchical composite adhesive structure consisting of thin plates, setae, and spatulas that can be adapted to the microscopic morphology of the wall surface and generate strong van der Waals forces by making intimate contact with the wall surface. Next, the controlled adhesion properties of the gecko were introduced in relation to the gecko’s macroscopic regulation of the toe muscles and the shear motion of the setae. Then, the design and preparation of gecko-inspired controllable adhesives were highlighted. Finally, we presented several important applications of gecko-inspired controllable adhesives.

**Figure 19:**
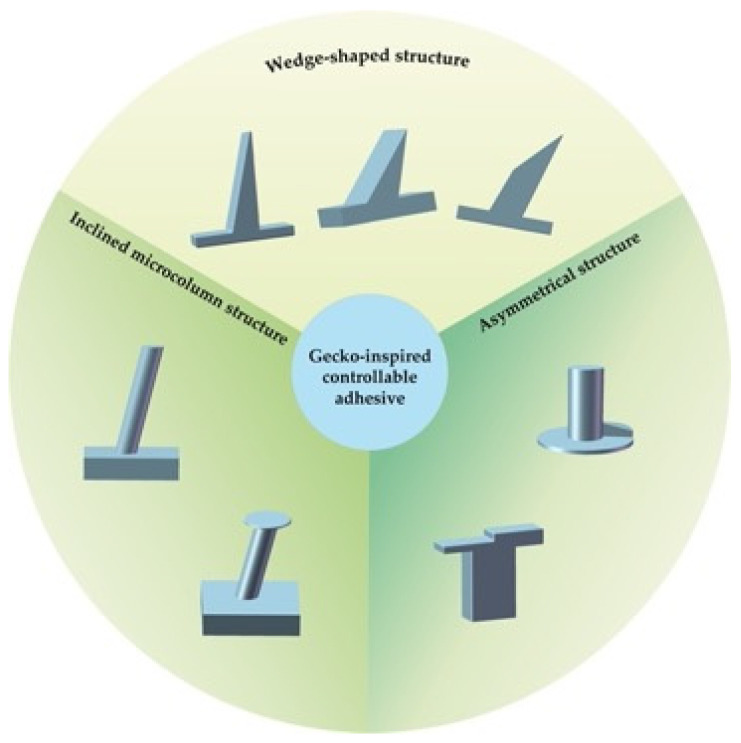
Typical design of gecko-inspired controllable adhesive microstructures.

As shown in Figure 19, for the structural design of gecko-inspired controllable adhesives, the design of anisotropic microstructures (such as wedges) is a direct means to achieve controllable adhesion. Adding tip features such as spatulas and mushrooms to the microstructures can improve the adhesion force. In addition, adding magnetizable particles in polymers, shape memory polymers as materials, other methods of preparing gecko-inspired controllable adhesives that can be in the magnetic field, temperature, and other active control methods to make the adhesive micro-geometry deformation or backing layer stiffness change, thus realizing the gecko-inspired adhesive state of active switching.

Gecko-inspired controllable adhesives have been developed for more than a decade, and their adhesion and controllable performance have been greatly improved, but there are still many challenges to fully reproduce the gecko’s strong wall-climbing ability. First, the current design for the microscopic geometry of gecko-inspired controllable adhesives is mostly determined by experience and intuition, and lacks a scientifically optimized structural design scheme to optimize the adhesion performance of the adhesion surface. Therefore, by combining machine learning and the design of adhesion surface microstructures, people can study the optimal design of an adhesive with more types of microstructures in the future. Second, the preparation efficiency of the existing gecko-inspired controllable adhesives is low, so how to realize the efficient preparation of large areas is an issue that should be considered in the future. Finally, the adhesion system of geckos is complex. Therefore, how to integrate all the adhesion mechanisms of the gecko into one product will be the future research direction of gecko-inspired controllable adhesives.

Gecko-inspired controllable adhesives have a wide range of applications in the field of climbing robots and robotic gripping tasks. However, existing gecko-inspired climbing robots are large compared to geckos in terms of their structural size and weight, and can only climb on smooth wall surfaces. The gripper’s grasping objects also have more than just smooth surfaces. Therefore, improving the self-cleaning, durability, and adaptability to rough wall surfaces of gecko-inspired controllable adhesives will be a long-term challenge.

## Figures and Tables

**Table 1:** Gecko-inspired controllable adhesives based on shear adhesion.

Material	Shape of Microstructure	Fabrication Method	Tested Area	Normal Adhesion	Shear Friction	Anisotropy Coefficient *
Polyurethane (IE-20 AH) [59]	Directional polymer stalks	UV lithography andmolding	3.9 cm^2^	1 N	8.0 Kpa	NA
Teflon [60]	Tilt nanohairs	Molding and e-beam irradiation	1 cm^2^	16 nN	110 Kpa	5
Polypropylene [62]	Tilted microcolumn	Molding and etching	4 cm^2^	NA	45 Kpa	45
Sylgard 184 [63]	Angled half-cylinder	Two-step photolithography and molding	12.6 mm^2^	NA	78 Kpa	6.2
Sylgard 170 [64]	Vertical wedge	Two-mask angled exposure and molding	1 cm^2^	5.1 Kpa	17.0 Kpa	NA
Sylgard 170 [65]	Tilted wedge	Micromachining process and molding	1.21 cm^2^	38 ± 2 Kpa	49 ± 1 Kpa	NA
Sylgard 184 [66]	Tilted wedge	Micromachining process and molding	2.84 mm^2^	10.5 Kpa	50 Kpa	1.67
Sylgard 184 [68]	Annular wedge	Ultraprecision machining and molding	NA	NA	35.48 mN	1.36
Polyurethane Acrylate [73]	Tilted nanohairs with flat tip	Etching and molding	3 cm^2^	NA	260 Kpa	11.8
ST-1060 [78]	Tilted mushroom tip	Photolithography and molding	1 cm^2^	NA	100 Kpa	5
NOA81 [79]	Tilted mushroom shape	UV photolithography and molding	1 cm^2^	NA	84 Kpa	2.4
Sylgard 184 [80]	Inclined quadrangles with rectangular tips	Two-step photolithography and molding	1 cm^2^	45 Kpa	55 Kpa	2.2
Sylgard 184 [81]	Tilted mushroom tip	2PP and molding	1 cm^2^	11.0529 ± 0.4093 Kpa	NA	7.52
Sylgard 184 [82]	Mushroom shape with stepped end	Two-step photolithography and molding	9 mm^2^	26 mN	NA	NA
Sylgard 184 [83]	Stem with mushroom structure of TPS	2PP and molding	NA	87.8 Kpa	NA	1254
ST-1060 [84]	Tilted trigonal with rectangular tips	Two-layer etch and molding	32 mm^2^	12.5 Kpa	28 Kpa	7.37

* Anisotropy coefficient: the ratio of the adhesion force in the direction of adhesion to the detachment force in the direction of detachment.

**Table 2:** Physical properties of common casting materials.

Material	Elastic Modulus (Mpa)	Tensile Strength (Mpa)	Viscosity (cp)	Hardness	Curing Type
ST-1060	2.9	6	NA	60 A	Ordinary
Sylgard 184	2.16 [113]	6.7	3500	43 A	Heater
Sylgard 170	1.95 [113]	3.7	2135	47 A	Heater

## Data Availability

Not applicable.
